# ResNet for recognition of Qi-deficiency constitution and balanced constitution based on voice

**DOI:** 10.3389/fpsyg.2022.1043955

**Published:** 2022-12-05

**Authors:** Tong Lai, Yutong Guan, Shaoyang Men, Hongcai Shang, Honglai Zhang

**Affiliations:** ^1^School of Medical Information Engineering, Guangzhou University of Chinese Medicine, Guangzhou, China; ^2^Key Laboratory of Chinese Internal Medicine of Ministry of Education and Beijing, Dongzhimen Hospital Affiliated to Beijing University of Chinese Medicine, Beijing, China

**Keywords:** Qi-deficiency constitution, balanced constitution, voice, ResNet, constitution in traditional Chinese medicine

## Abstract

**Background:**

According to traditional Chinese medicine theory, a Qi-deficiency constitution is characterized by a lower voice frequency, shortness of breath, reluctance to speak, an introverted personality, emotional instability, and timidity. People with Qi-deficiency constitution are prone to repeated colds and have a higher probability of chronic diseases and depression. However, a person with a Balanced constitution is relatively healthy in all physical and psychological aspects. At present, the determination of whether one has a Qi-deficiency constitution or a Balanced constitution are mostly based on a scale, which is easily affected by subjective factors. As an objective method of diagnosis, the human voice is worthy of research. Therefore, the purpose of this study is to improve the objectivity of determining Qi-deficiency constitution and Balanced constitution through one’s voice and to explore the feasibility of deep learning in TCM constitution recognition.

**Methods:**

The voices of 48 subjects were collected, and the constitution classification results were obtained from the classification and determination of TCM constitutions. Then, the constitution was classified according to the ResNet residual neural network model.

**Results:**

A total of 720 voice data points were collected from 48 subjects. The classification accuracy rate of the Qi-deficiency constitution and Balanced constitution was 81.5% according to ResNet. The loss values of the model training and test sets gradually decreased to 0, while the ACC values of the training and test sets tended to increase, and the ACC values of the training set approached 1. The ROC curve shows an AUC value of 0.85.

**Conclusion:**

The Qi-deficiency constitution and Balanced constitution determination method based on the ResNet residual neural network model proposed in this study can improve the efficiency of constitution recognition and provide decision support for clinical practice.

## Introduction

Constitution refers to the inherent characteristics that are comprehensive and relatively stable in the morphological structure, physiological function, and psychological state formed on the basis of congenital endowment, and are acquired in the process of human life, and categorized into nine types (Li et al., 2019). A Balanced constitution is a relatively healthy constitution, while a Qi-deficiency constitution is a constitutional state characterized by low breath and low functions of the body and the zang-fu organs due to a deficiency of the primordial Qi (Wang, 2005). People with a Qi-deficiency constitution tend to have a lower voice frequency, shortness of breath, reluctance to speak, introverted personality, emotional instability and timidity and are prone to repeated colds and have a higher probability of chronic diseases (Wang, 2019). The survey showed that the Qi-deficiency constitution of the Chinese population ranked second in the biased constitutions (Bai et al., 2020), and ranked in the top three in obstructive sleep apnoea-hypopnea syndrome (Xu et al., 2022), irritable bowel syndrome (Yin et al., 2022), primary dysmenorrhea (Bi et al., 2022), and depression (Yang et al., 2016).

At present, the determinations of Qi-deficiency constitution and Balanced constitution are mostly based on the classification and assessment of TCM constitutions (China Association of Traditional Chinese Medicine, 2009) but this measurement method is based on a questionnaire, and is time consuming and easily influenced by various subjective and objective factors, which leads to inaccurate survey results (Yan et al., 2014). Therefore, it is necessary to use more convenient and objective measurement methods to identify TCM constitutions in a more flexible and efficient way. Acoustic diagnosis is one of the important tasks of auscultation, which refers to a method that uses hearing to diagnose diseases. Information is easily acquired *via* hearing, and many studies have proven that speech can be used to diagnose diseases (Jin et al., 2015; Tjaden and Martel-Sauvageau, 2017; Vaiciukynas et al., 2017; Taguchi et al., 2018; Wang et al., 2019). With the development of artificial intelligence and data mining methods, some studies have begun to use machine learning to classify audio tasks. Shi et al. (2008) used an HMM classifier to make an initial audio classification and then used an SVM classifier to determine the audio category, which effectively classified standardized audio types such as speech and music with high accuracy. Lu and Hankinson (1998) used the idea of a decision tree to classify different audio types so that the machine could distinguish between human voice and noise, and also understand the emotion and speed in the audio.

Machine learning can correctly classify music, voice and noise. This kind of algorithm can classify these samples according to eigenvalues such as the zero-crossing rate and the pitch frequency of different audio in the same classification. However, with the increase in the amount of data and the requirements of deep of learning, the number of features that need to be extracted and used for classification increases gradually. Because the feature information that machine learning models can learn is limited, an increasing number of researchers tend to use deep learning models to accomplish audio-related classification tasks. Xiong et al. (2021) compared the advantages and disadvantages of five models, namely, SVM, Bayesian classifier, fully connected network, convolutional neural network (CNN) and recurrent neural network (RNN), and found that CNN was the ideal model for audio classification. At present, CNNs have been widely used in image, voice and other fields, and various networks modified on the basis of CNNs are widely used because of their powerful functions. At the same time, some studies have proven that higher classification accuracy can be obtained when machine learning and deep learning models are combined. Fu and Yang (2018) proposed associating CNN with random forest for audio classification, which exert the characteristics of random forest ensemble learning for audio classification. Wu et al. (2021) used the collocation of KNN (k-nearest neighbor algorithm) and CNN to classify the audio for the hearing of customer service calls, which can be used to process users’ calls and classify the reasons for the calls. These studies have effectively improved the accuracy of audio classification but these deep learning models are only applied to the field of audio classification, and do not accomplish the classification of constitutions by voice. In terms of using voice recognition for constitution classification, Yong and Xu (2013) et al. compared the seven sound parameters including parameters pitch, pitch jitter, shimmer, formant variation, zero-crossing rate ratio, the voice sound energy duration, and area of speech envelope parameters, of the young voices of Qi-deficiency constitution and Balanced constitution subjects, and they found that there were significant differences in the two parameters. In addition, Yong et al. (2016) also analyzed the pitch jitter and amplitude perturbation of young people’s voices of Qi-deficiency constitution and Balanced constitution subjects through the sound disturbance analysis method and found that the stability of the Balanced constitution subjects was better than that of the Qi-deficiency constitution subjects. Su et al. (2013) found that the maximum pitch of the subjects in the high-Qi-deficiency constitution group was higher than that in the low-Qi-deficiency constitution group by extracting the voice parameter of vowel /a/ uttered by the subjects after the subjects with a constitution score higher than 40 were divided into the high group, and those with a constitution score lower than 40 were divided into the low group. Mu (2012) extracted the LPCC and MFCC of Yi He in subjects with a Qi-deficiency constitution and a Balanced constitution and applied the SVM and BP neural network to classify the Qi-deficiency constitution and Balanced constitution. The results showed that the use of LPCC and MFCC could distinguish between the two constitutions to a certain extent. Li et al. (2022) reviewed the related studies on constitution recognition using voice, and proposed that a voice diagnosis model could be established for constitution recognition. However, the existing research only uses the method of comparing the relevant voice parameters, and also uses machine learning, to identify the constitution, and does not use the deep learning method to establish a voice diagnosis model of the constitution. During the learning process of a deep neural network, due to the gradual increase in data and the superposition of the model layers, a gradient explosion and gradient disappearance phenomenon will occur due to the nonlinear calculation of the features, thus weakening the learning ability of the network, and the classification results will deteriorate.

To solve the above problems, in this study, we proposed using the ResNet (He et al., 2016) network model to analyze the subject’s voice to better identify Qi-deficiency constitution and Balanced constitution. ResNet resolves the degradation phenomenon that easily occurs when the layers of the convolutional neural network are deep. It can establish a deeper convolutional neural network model, thus enhancing the learning ability of the network. Therefore, in this study, the voices of populations with Qi-deficiency constitutions and Balanced constitutions were collected, and the voices were converted into frequency spectra and sent to the ResNet deep learning model for feature learning to ascertain the subject’s constitution type. At the same time, according to the working curve (ROC) of the subjects, the accuracy of reasonable classifications of Qi-deficiency constitution and Balanced constitution were evaluated.

## Materials and methods

### Study subjects

All the subjects were recruited from college students and graduate students at Guangzhou University of Traditional Chinese Medicine. The inclusion criteria were that the subjects needed to be over 18 years old, and the exclusion criteria were no subjects were used that suffered from acute diseases or acute pain, or who took any drugs in the past 3 months. This study was approved by the Ethics Committee of Traditional Chinese Medicine Hospital of Guangdong Province (YF2022-037-01). All the subjects signed an informed consent form before the experiment.

### Constitution judgment

All the subjects were judged as having a Qi-deficiency constitution and a Balanced constitution by the TCM Constitution Scale (China Association of Traditional Chinese Medicine, 2009) published by the Chinese Society of Traditional Chinese Medicine in 2009. The internal consistency of the TCM Constitution Scale is between 0.72 and 0.82, and the retest reliability is between 0.77 and 0.90 (Zhu et al., 2006). Each constitution in this questionnaire includes six to eight questions, and the answer to each question is evaluated according to the five-point Likert scale (Never = 0, Occasionally = 1, Sometimes = 2, Often = 3, and Always = 4). Then, the original total score is converted to 0–100 points, and some of the subjects with abnormal constitution scores over 40 points were identified as having a certain type of constitution.

### Voice acquisition

This study collects two kinds of voice content based on a literature survey (Chen et al., 2010; Hu, 2014; Dong et al., 2015; Song et al., 2019; Chen, 2021). The first is the vowel (the first tone of a Chinese syllable) “a, o, e, i, u.” The literature (Hu, 2014; Dong et al., 2015; Chen, 2021) uses vowels to analyze the voices of patients with lung diseases, and the literature (Chen et al., 2010) uses vowels to analyze the voices of patients with deficiency syndrome and excess syndrome as defined in traditional Chinese medicine. Another kind of voice content is the words “Hai (sea), Yun (cloud), Xin (heart), Shi (Ten), Kou (Mouth), Yue (Moon), Dong (East), Ni (Mud), Fang (Square) and Mei (Beauty).” The literature (Song et al., 2019) used words for an objective analysis for an acoustics diagnosis.

The sound of the voices were collected in a quiet room, and the ambient noise was less than 35 dB. During the acquisition process, the subjects were required to relax and sit in a natural and comfortable position. The microphone model was a AKG HSC271, its signal-to-noise ratio was 22 dB, and the distance between the mouth of the subjects and the microphone was approximately 5 cm. Praat 6.1.32^1^ software was used to collect the sound, the sampling frequency was 44,100 Hz, and the channel was mono.

### Data processing

**Figure 1:**
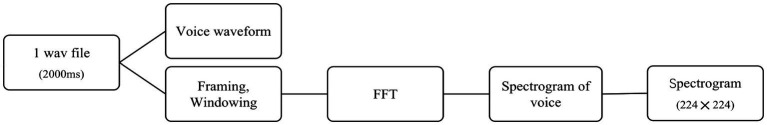
The process of voice pre-processing.

Frequency spectrograms are usually used to process tasks related to speech analysis, such as voice recognition (Hou et al., 2020) and voice emotion analysis (Tursunov et al., 2021). Its horizontal axis is time, and its vertical axis is frequency. It shows the changes in the signal intensity of the different frequencies displayed by different sounds with time. We adopt the method of this article (Tursunov et al., 2021) for generating the spectrogram. As shown in Figure 1, the collected voices are saved in the wav format, and the data are processed in the next step. Before generating the spectrogram, on the premise of ensuring the integrity of the recorded content, we cut the lengths of all the voices to 2000 ms, obtain the voice waveform and then create a frame and window of each voice. Next, a fast Fourier transform (FFT) is performed on each frame to obtain the spectrogram corresponding to each voice. In the end, the size of each spectrogram is 224 × 224 when it is input into the model. The processed spectrograms are randomly divided into a training set and testing set according to proportions of 85 and 15%, which are then used for the training and testing of the model to judge whether the voice master belongs to a Qi-deficiency constitution or a Balanced constitution.

**Figure 2:**
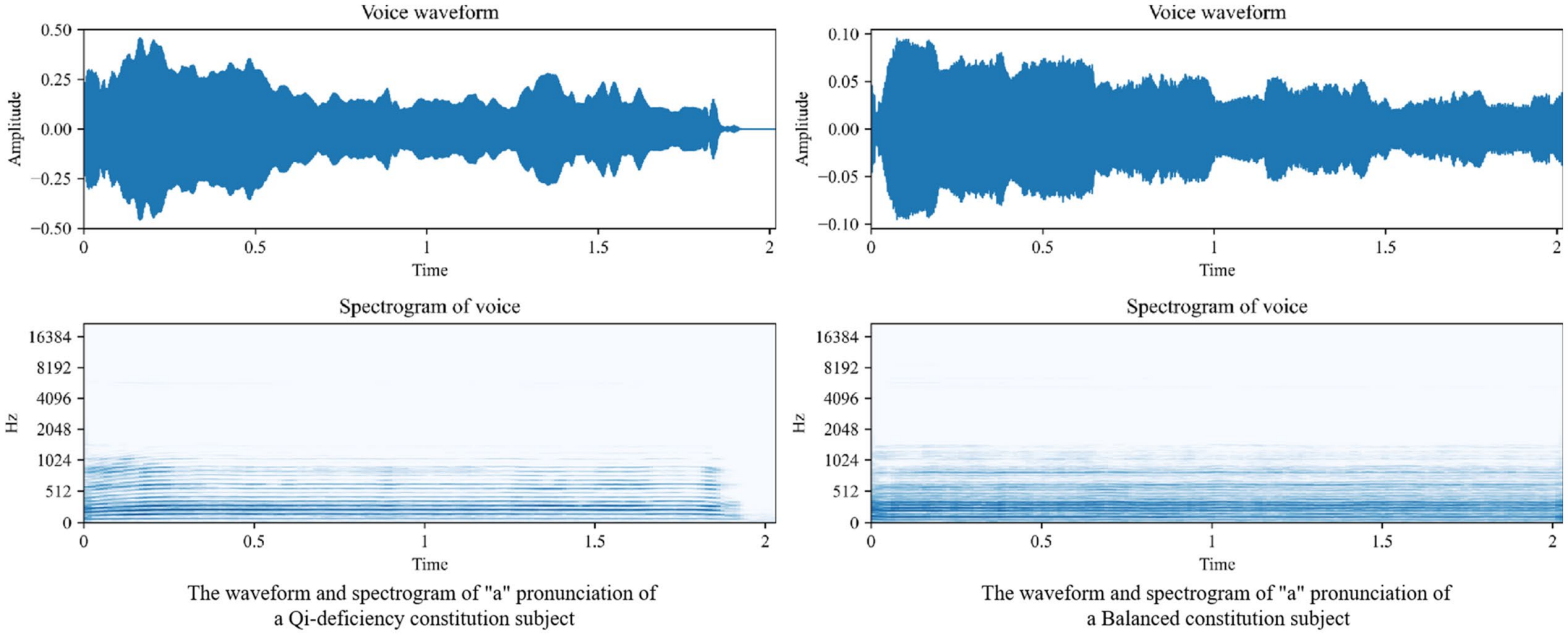
voice waveform and spectrogram of Qi-deficiency constitution and Balanced constitution subject.

Figure 2 shows the voice waveform and spectrogram of the different voices; the left shows the voice waveform and spectrogram of the “a” pronunciation of a Qi-deficiency constitution subject, and the right shows the voice waveform and spectrogram of the “a” pronunciation of a Balanced constitution subject.

### Model construction

**Figure 3:**
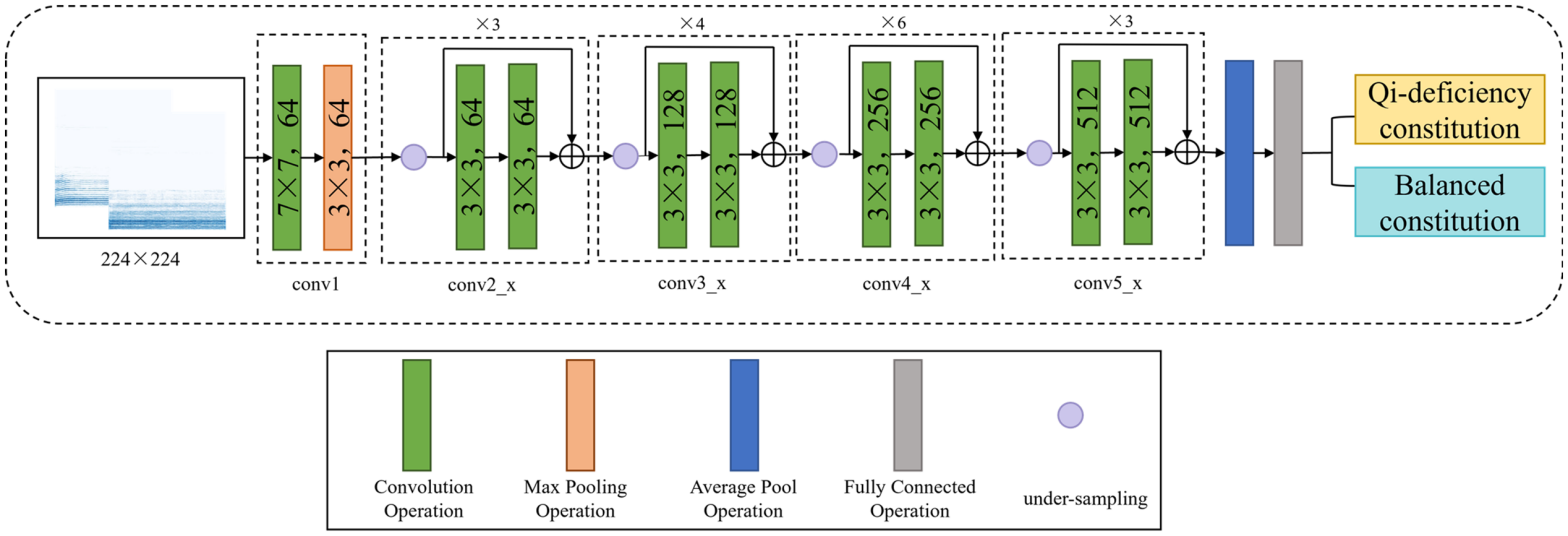
Structure framework of constitution classification model using ResNet network.

In this study, the residual neural network model based on ResNet (Su et al., 2013) is used to classify the collected voice data. The ResNet family mainly includes ResNet18, ResNet34, ResNet50, ResNet152, etc. After the previous experiments, ResNet34 with better results was used in the experiment in this study. Figure 3 shows the model framework of this study.

In this experiment, we use two convolution layers of the convolution kernel size. The preprocessed spectrogram is first sent to the first convolution layer, with the number of input channels set to 3, the number of output network channels set to 64, the size of the convolution kernel set to 7, the step size set to 2 and the padding set to 3, to implement the convolution operation for two-dimensional images. Then, through a layer of Maxpool, the spectrogram is divided into a number of large blocks according to requirements, and the maximum value is extracted from each feature to represent the block. After discarding the other features, the output is obtained. In this experiment, the maximum pool kernel size is 3, the step size is 2 and the filling is 1. Conv2_x, conv3_x, conv4_x, and conv5_x are the four convolution layers containing the residual structures with the same convolution kernel size but different channel values. By controlling the number of input channels and the size of the convolution kernel, the required extraction features are selected, and a corresponding mapping is calculated to obtain the final output.

**Figure 4:**
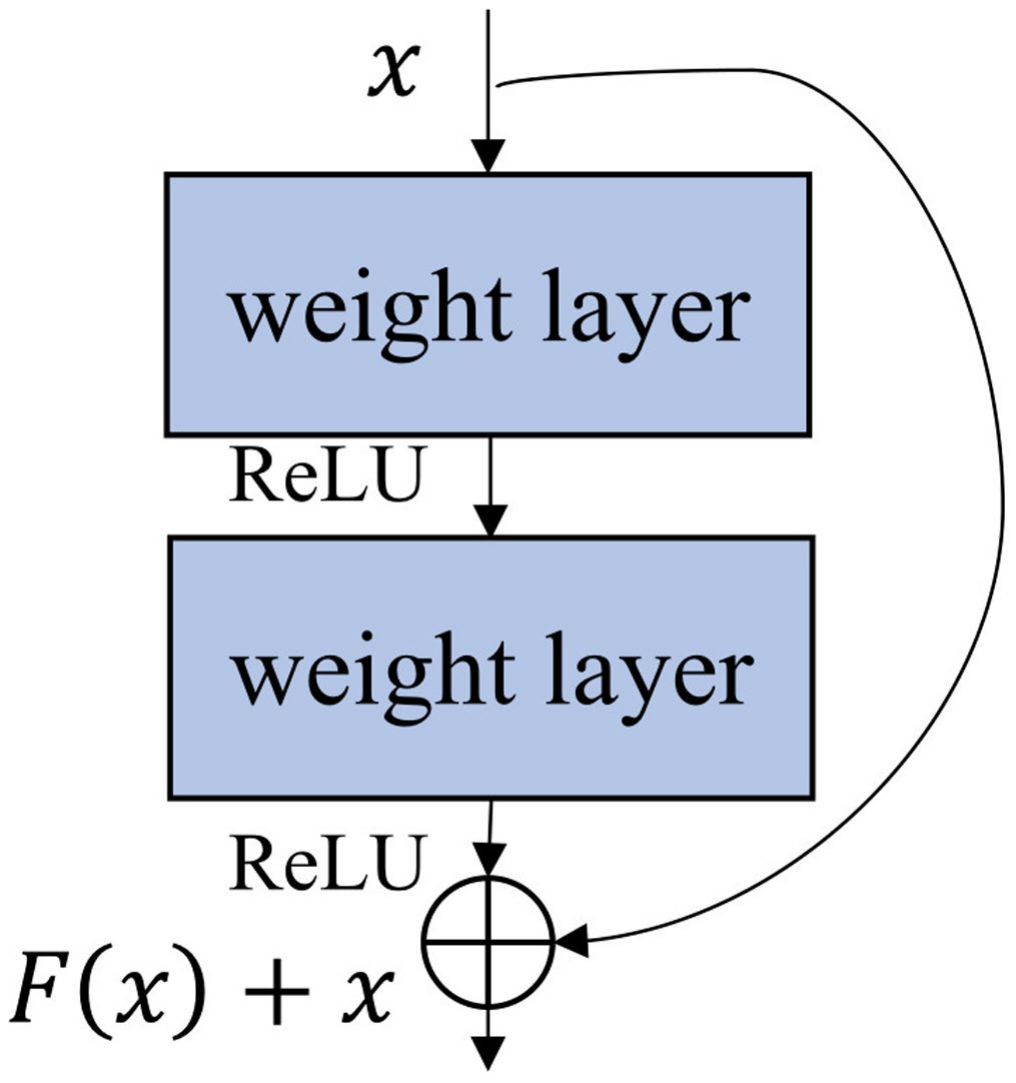
Single shortcut connection.

To obtain better classification results, the depth network needs to learn more accurate features, and the depth of the network plays a very important role in feature discovery. The deeper the network is, the stronger the features that can be found, making the classification effect more accurate. However, in the process of data learning, the more network layers that are built, the higher the accuracy of the results. This is because, with an increase in network layers and the deepening of learning, the number of nonlinear transformations (such as the activation functions) used is constantly increasing, and no identity mapping can be found, so the growth error of this layer increases and “degradation” occurs. To avoid this phenomenon and its impact on the network, ResNet adds a shortcut connection branch to the module, as shown in Figure 4, to record the errors of the lower layer and pass them to the upper layer (Zhou et al., 2022). This residual module can effectively reduce the number of direct gradient passes in the backpropagation of the neural network, allowing the network to perform a more complete feature learning. Compared with y = F(x), y = F(x) + x is easier to optimize. This method substantially eliminates the inaccurate prediction caused by the increase in the network layers and resolves the difficult problem of neural network training with excessive depth.

**Figure 5:**
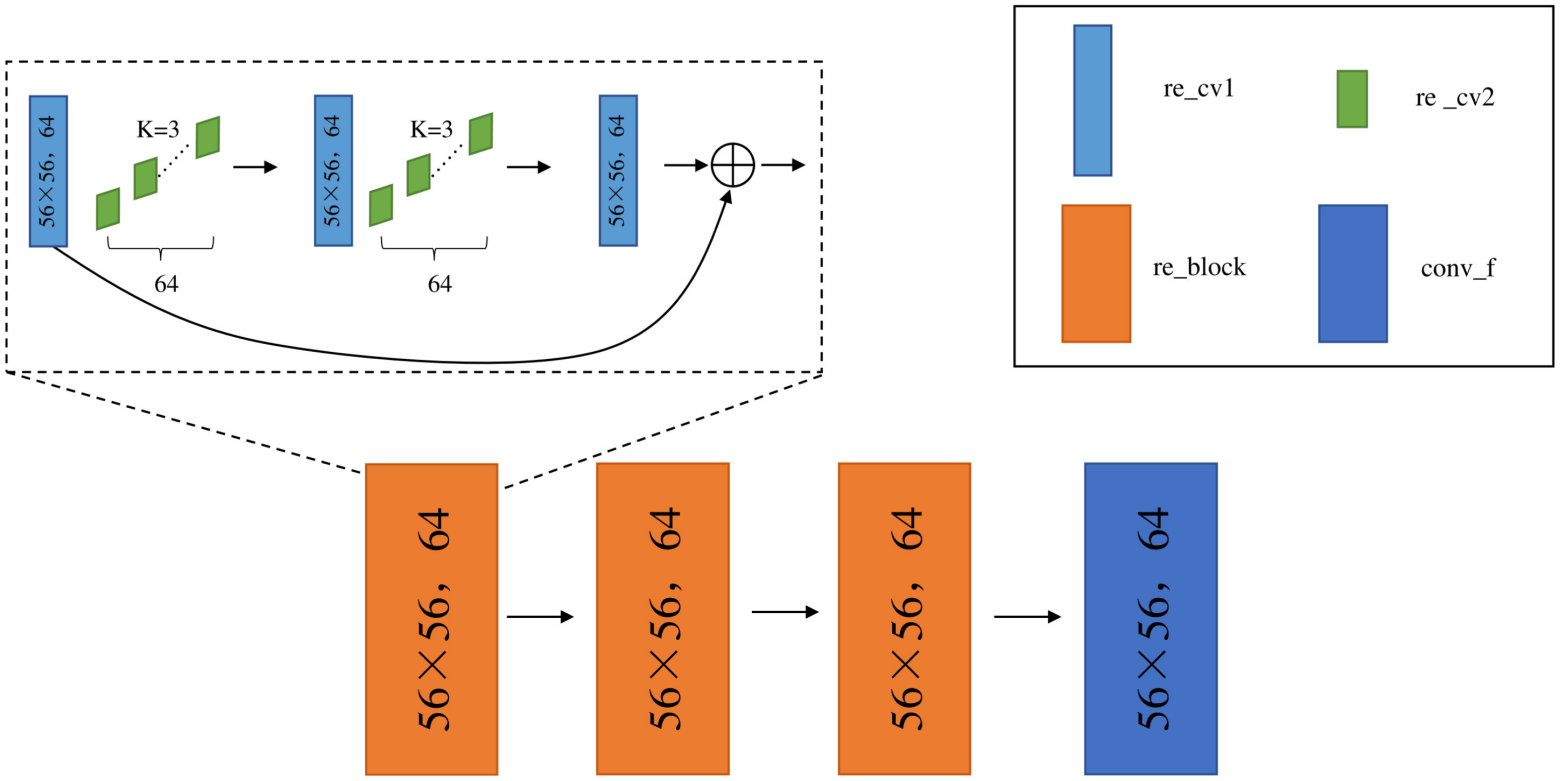
network structure of Conv2 _ x layer.

In this experiment, the residual module is added into the middle four layers based on the ResNet34 network model, as shown in Figure 5. Take the first convolutional layer with residual blocks “conv2_x” as an example. “re_cv1” is the convolutional network. A convolutional operation is performed on the image using 64 convolutional kernels “re_cv2” of size 3 × 3. Then, this structure is repeated, and the first layer network feature values are summed with the last layer network feature values (i.e., the features of the deep network and the shallow network) when the result is returned to obtain the network output “re_block” after the residual. There are three such residual structures in conv2_x specified in ResNet34, so the above steps are repeated three times to obtain the network output conv_f with the residual structures in the first layer.

The times of using the residual structure are 3, 4, 6, and 3; that is, there are 16 residual structures in this network. After the residual structure is used to reduce the deviation caused by the depth network, ReLU is used as the activation function for backpropagation and gradient descent to carry out multiple rounds of learning. After extracting the maximum feature value with Maxpool, the input tensor size is changed with the full connection layer, and the final binary classification result is obtained. Table 1 shows the ResNet34 model parameters. It shows the number of layers and the output size for adding the residual structures in the different convolutional layers, and illustrates the normalization method.

**Table 1:** ResNet34 Model Parameters.

Layer name	Output size	34-layer
conv1	112 × 112	7 × 7, 64, stride 2
conv2_x	56 × 56	3 × 3 Maxpool, stride 2
		$\left[\begin{array}{cc} 3\times 3 & ，64\\ 3\times 3 & ，64 \end{array}\right]$ ×3
conv3_x	28 × 28	$\left[\begin{array}{cc} 3\times 3 & ，128\\ 3\times 3 & ，128 \end{array}\right]$ ×4
conv4_x	14 × 14	$\left[\begin{array}{cc} 3\times 3 & ，256\\ 3\times 3 & ，256 \end{array}\right]$ ×6
conv5_x	7 × 7	$\left[\begin{array}{cc} 3\times 3 & ，512\\ 3\times 3 & ，512 \end{array}\right]$ ×3
average pool, 2-d fc, softmax		

### Evaluation index

In this study, we use learning accuracy (ACC), ROC, and AUC to evaluate the final result, and we calculate the difference between the predicted classification and the real classification by a loss value. ACC can directly show the performance of the model, and the performance of the model can be obtained by calculating the difference between the predicted value and the true value by the loss function. The larger the loss value is, the larger the gap between the predicted value and the real value, and the model needs to be continuously optimized. In the training process, under normal circumstances, the loss value will generally show a downward trend. When learning features and classifying them according to voice features, care should be taken to avoid overfitting and underfitting. If the model in the training set performs well with good results, but in the test set, the same model does not perform well enough or too poorly, it is usually necessary to consider whether the generated model is overly adapted to the training set data, resulting in poor generalization, and thus cannot be applied to the test set or other datasets. There are many reasons for this situation; for example, the model could be too complex, and the samples may not be representative and diverse. Underfitting is the opposite of overfitting, where the model is too simple and the number of features is too small, resulting in the model not being able to learn the patterns in the datasets, and thus the phenomenon of underfitting occurs.

To monitor the learning efficiency and the final results of the model in real time, and to avoid the overfitting and underfitting problems, the difference between the loss value (loss-train) of the training set and the loss value (loss-test) of the test set can be compared to determine whether the model is overfitting or underfitting during the learning process. If the loss-train is lower but the loss-test is higher, the model has been overfitted in the training process, and the model cannot evaluate the test set data correctly because it is too suitable for the training set data. If the loss-train drops gently, it means that the model has not learned useful features, so the problem of underfitting appears.

The ROC curve can reflect the relationship between sensitivity and specificity. For a binary classification problem, the two categories can be divided into positive category 1 and negative category 0. The X-axis (false-positive rate, *FPR*) in the ROC curve is 1-specificity (1−*TNR*), which indicates that the proportion of data whose actual label is 0 but predicted to be 1 in all the actual labels is 0. The Y-axis (true positive rate, *TPR*) is the sensitivity, which indicates the proportion of the data whose actual label is 1, and is correctly predicted in the total number of all the actual labels with 1. The formula is as follows:


(1) $$TPR=\frac{TP}{P}=\frac{TP}{TP+FN}$$

(2) $$FPR=\frac{FP}{N}=\frac{FP}{FP+TN}=1-TNR$$

where *TP* is the number of actual tags being 1 and predicted to be 1, *FN* is the number of actual tags being 1 but predicted to be 0, *FP* is the number of actual tags being 0 but predicted to be 1, and *TN* is the number of actual tags being 0 and predicted to be 0.

The AUC is the area formed between the ROC curve and the X-axis, and the size of the area is generally a number between 0 and 1. If the AUC value is greater than 0.5, it is generally considered that the experiment is meaningful. At the same time, the closer the area is to 1, the easier it is to prove the authenticity of the experiment.

The loss function we use is *CrossEntropyLoss*, which is a cross-entropy loss and is usually used for classification problems. *CrossEntropyLoss* does not take the probability distribution of the output as the basis of the classification but is more concerned with the similarity between the predicted classification and the real results. What it needs to do is to close the distance between them, that is, minimize the cross entropy, which can improve the learning accuracy of the model.

## Results

### Subject characteristics

A total of 720 voices of 48 subjects were enrolled, of which 20 were had a Balanced constitution and 28 were Qi-deficiency constitution subjects, both groups were aged 18–23 years. From the gender point of view, there are 11 males and 9 females with Balanced constitutions; there are 13 males and 15 females with Qi-deficiency constitutions. Table 2 shows the sex, age and average time to complete the questionnaire of the subjects.

**Table 2:** Characteristics of subjects.

Characteristics	Value
Male	24 (50.0%)
Female	24 (50.0%)
Age (year)	19.62 ± 1.28 (18–23)
Questionnaire completion time(s)	480.16 ± 180.62 (423–716)

Data are shown as Mean ± SD (minimum–maximum) for continuous variables and count (percentage) for categorical variables.

### Model results

To ensure the best performance of the model, the relevant parameters in the model need to be determined. The finalized values of each parameter based on experience are shown in Table 3. The input image size of the model is 224 × 224, and the initial values of the RGB channels are 0.5, 0.5, and 0.5. To achieve the best results for the model, we adopt a control variable, and the experiments show that the learning rate, step size, and multiplication factor of the learning rate of the model were 0.0001, 30, and 0.99, respectively.

**Table 3:** Model parameter settings of ResNet34.

Parameters	Value
Learning epoch	50
Initial image size	224 × 224
RGB initial normalized values	(0.5, 0.5, 0.5)
Batch size	8
Learning rate	0.0001
Step size	30
Multiplication factor of learning rate	0.99

**Figure 6:**
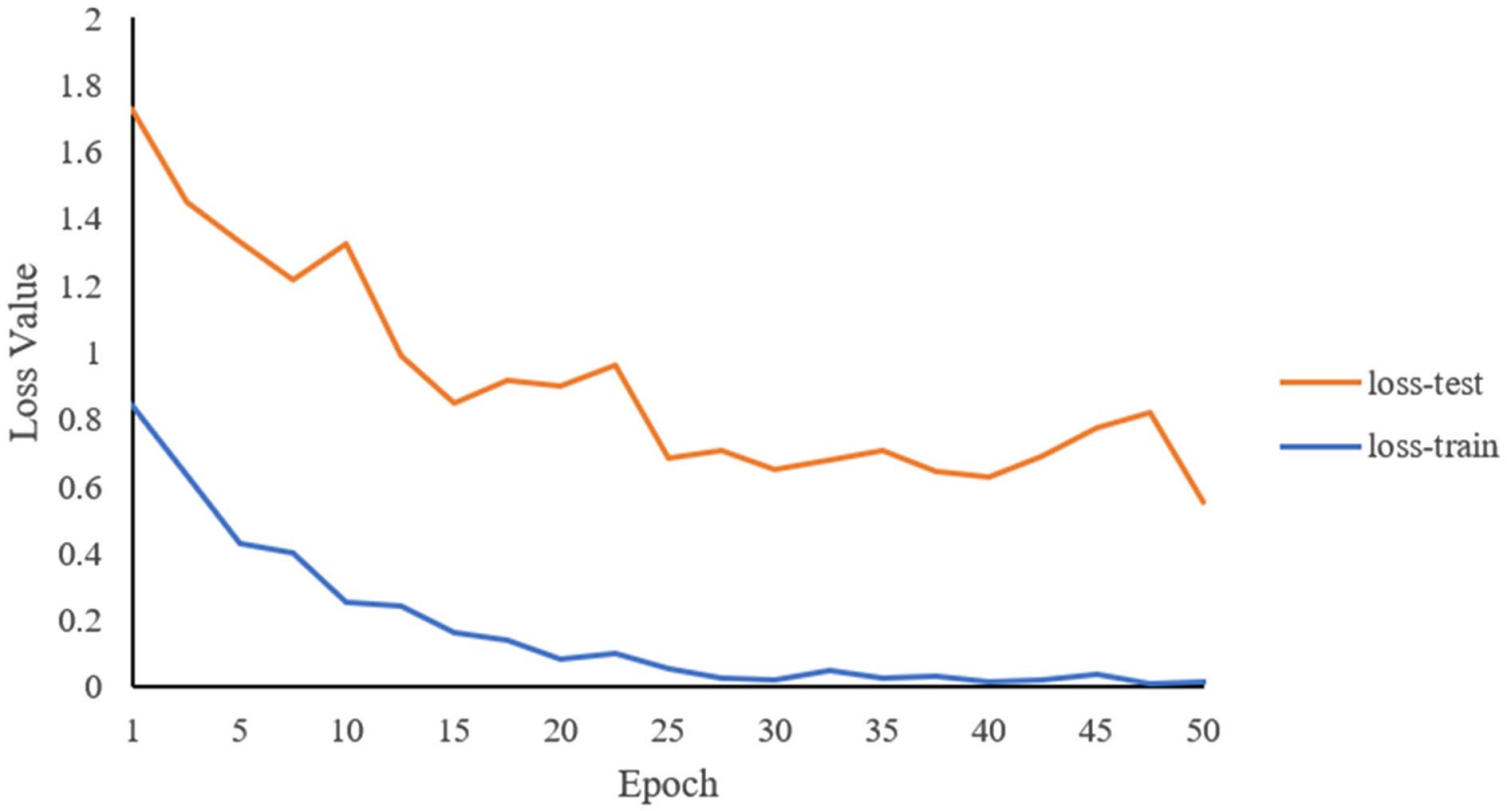
Changes of loss values of training set and test set with the increase of learning rounds.

**Figure 7:**
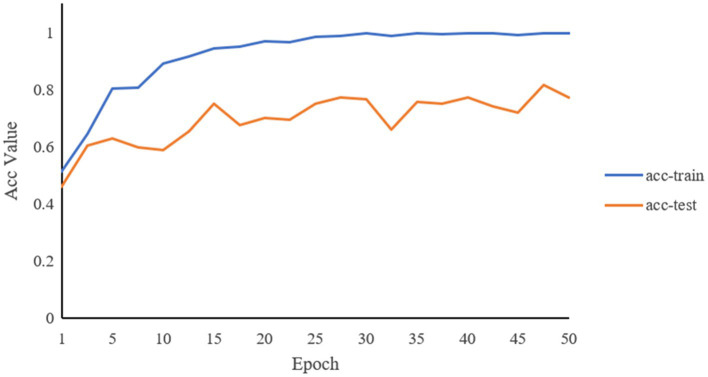
Changes of ACC values of training set and test set with the increase of learning rounds.

**Figure 8:**
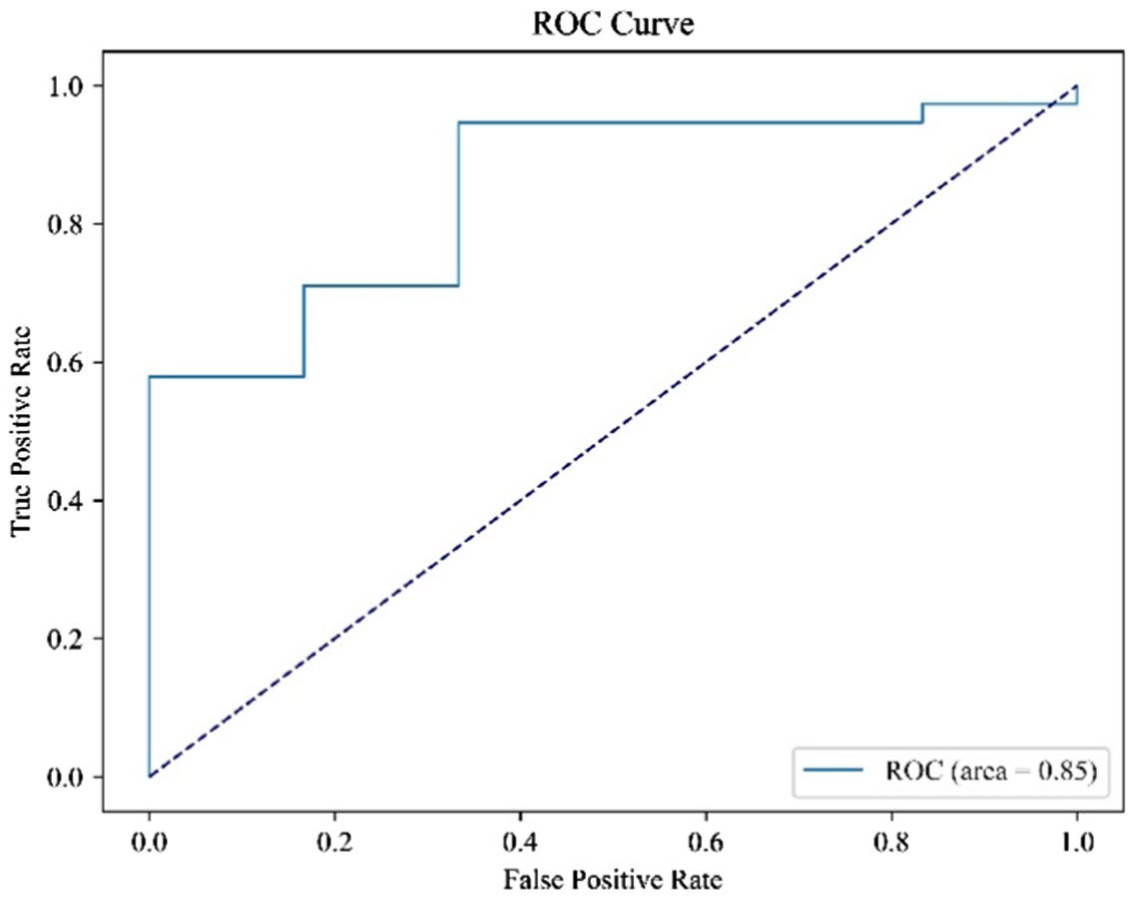
ROC curve after using ResNet network.

Through the predictions of the abovementioned deep learning network, the accuracy of the classifications of the two constitutions is 81.5%. Figures 6, 7 record the changes in the loss value and ACC value, respectively. In Figure 6, with the increase in the number of learning rounds, the loss values of both the training set and the test set gradually decreased and tended to be 0, indicating that the model has not caused an overfitting or underfitting in the learning process. The ACC values of both the training and the test sets in Figure 7 showed an increasing trend, and the ACC value of the training set is close to 1. This indicates that the model has learned sufficiently for the data contained in the training set, and there is no overfitting. The model, through learning, is able to accurately predict the classification of the data in the test set and determine what kind of human body the language data pertain to. The ROC curve is shown in Figure 8, and the AUC value of the curve is 0.85.

## Discussion

From the TCM constitution theory perspective, a Balanced constitution is in a state of harmony between the yin and yang, as well as between the qi and blood, indicating that the person is in a healthy state. However, people who have a biased constitution are subject to certain diseases. The traditional Chinese medicine theory holds that qi is the basic material used to maintain the physiological activities of the human body, and a Qi-deficiency constitution is likely to cause dysfunctions of the internal organs in the body. Studies on the correlation between constitutions and diseases have found that a Qi-deficiency constitution is indicated in a higher proportion of patients with stroke, diabetes, COPD, AIDS, and hypertension (Liang et al., 2020). Additionally, people with a Qi-deficiency constitution are introverted and do not like adventure. This is because people with Qi-deficiency may have the characteristics of cowardice. Lv et al. (2022) analyzed the correlation between nine constitutions of 913 college students and 16 personality factors and found that the Qi-deficiency constitution had personality characteristics such as emotional excitement, fear, anxiety, tension, and distress. Thus, people with Qi-deficiency constitution are prone to physical and psychological problems.

At present, the determination of a Qi-deficiency constitution is mostly based on the classification and judgment results of the TCM constitution. This approach is prone to a subjective bias of the subjects, which increases the workload of the clinicians and affects their diagnosis and treatment efficiency. Therefore, some scholars propose identifying the constitution using objective signals (such as the voice). As a relatively objective and easily obtained indicator, pronunciation has certain advantages (Alghowinem et al., 2018). Mu (2012) selected Chinese characters such as Yi He that reflect the five notes of the ancient Chinese pentatonic scale as the recording content, and took MFCC and LPCC as acoustic indices to analyze the voice characteristics of college students with nine constitutions, and found that MFCC and LPCC could distinguish between the different constitutions to some extent. Sun et al. (2012) analyzed nine sound characteristics, such as pitch, range and the formant, the of vowel /a/ spoken by adults of nine different constitutions and found that the adult sound characteristics of the six constitutions, including the Qi-deficiency constitution, had significant differences. Huang et al. (2019) extracted the tongue, voice and pulse information of 274 subjects, and the related indicators, to construct a constitution recognition model, and found that the Qi-deficiency constitution had a negative correlation with the parameters related to sound intensity. It can be seen from the above that there is a certain research basis for the use of voice to identify constitutions.

With the development of artificial intelligence technology, CNNs have been widely used in the voice field. Therefore, this study adopts the deep learning method to explore the predictive function of pronunciation on Qi-deficiency constitution and Balanced constitution. In this study, we found that it is feasible to distinguish between the Qi-deficiency constitution and Balanced constitution using neural networks in the form of deep learning models, which learn information about the features of the two human body qualities and classify the samples based on those features. For the choice of algorithm, we used the ResNet network. As the network deepens, the model degenerates due to the nonconstant mappings resulting from redundant network layers. The most notable feature of ResNet is the addition of a residual mechanism to the convolutional neural network. This feature effectively mitigates the negative effects of gradient explosion or disappearance, and ensures that the model can obtain higher accuracies as a way to improve the overall performance of the network.

The experimental results show that a classification model based on the deep learning of voice can accurately determine whether an individual has a Qi-deficiency constitution or a Balanced constitution, and its classification accuracy rate is 81.5%. Based on the ROC curve, it can be seen that the method adopted in this study can achieve high results, which indicates the potential value of voice, which is an objective and easy-to-obtain data point, in quickly identifying Qi-deficiency constitution and Balanced constitution, improving the efficiency of constitution identification, reducing subjective deviation and laying a foundation for other research on biased constitution in the future. The conclusion that phonetics can predict Qi-deficiency constitution did have phonetic abnormalities. At the same time, it can help clinicians quickly determine information about patients’ constitution to develop different treatment methods accordingly.

There are the following deficiencies in this study. Since the main purpose of this study was to explore the feasibility of deep learning in speech recognition of Qi-deficiency constitution and Balanced constitution, the diversity of the population was not considered when the subjects were recruited, and the voices of the men and women were not separately investigated. In the future, we will expand the data volume to include people of all ages, and separately analyze the respective voice characteristics of the men and women to exclude the effect of potential confounding variables, and to improve the reliability and efficiency of the model in identifying Qi-deficiency constitution and Balanced constitution. There are nine types of constitutions, including one normal constitution and eight biased constitutions. Before the experiment, we decided that if the eight biased constitutions were included together, the phonetic features would be multifarious, and there was a particular peculiarity related to the sound in the manifestation of Qi-deficiency constitution (such as low voice, shortness of breath and no desire to speak). Therefore, the Qi-deficiency constitution and Balanced constitution were chosen as the research subjects in this study.

From the perspective of the algorithm, the ResNet34 network model used in this paper is able to distinguish between the two corpora but the model itself still has shortcomings. First, the computational speed of the model decreases as the amount of data increases. Therefore, improving the training speed of the model is a problem that needs to be solved. Second, the convolutional neural network can acquire features from spatial and channel information and learn them effectively but due to the limitation of the network structure, it cannot make full use of both types of information. Therefore, the model needs to use the attention mechanism to enhance the intensity of learning information from these two aspects to improve the accuracy of model classification. In future research, we will comprehensively examine the differentiation effect of the speech predictions of the nine body types in different genders and populations to form a better model to obtain more of the learning features hidden in the speech and implement the classification predictions of the nine body types of speech.

## Conclusion

In this study, a deep learning method is proposed to recognize Qi-deficiency constitution and Balanced constitution. The voices are processed into a spectrogram and then sent to the ResNet network model for feature learning, which can effectively distinguish the difference between Qi-deficiency constitution and Balanced constitution and then recognize them. In future research, more mature models can be used to differentiate the constitutions of more diverse types of people, thus assisting doctors in making constitution judgments more quickly.

## Data availability statement

The raw data supporting the conclusions of this article will be made available by the authors, without undue reservation. The code of this manuscript has been put on https://github.com/GYT0704/Voice_Classification.git.

## Ethics statement

The studies involving human participants were reviewed and approved by the Ethics Committee of Traditional Chinese Medicine Hospital of Guangdong Province (YF2022-037-01). The patients/participants provided their written informed consent to participate in this study.

## Author contributions

TL formulated the research method, performed the analysis, participated in the acquisition of data, and drafted the manuscript. YG did the experiment and drafted the manuscript. SM, HS, and HZ provided thesis guidance, administrative and material support. All authors contributed to the scientific discussion of the data and of the manuscript.

## Funding

This work was supported by the National Key Research and Development Plan (2019YFC1710402).

## Conflict of interest

The authors declare that the research was conducted in the absence of any commercial or financial relationships that could be construed as a potential conflict of interest.

## Publisher’s note

All claims expressed in this article are solely those of the authors and do not necessarily represent those of their affiliated organizations, or those of the publisher, the editors and the reviewers. Any product that may be evaluated in this article, or claim that may be made by its manufacturer, is not guaranteed or endorsed by the publisher.

## Footnotes

^1^ https://www.fon.hum.uva.nl/praat/
